# Recent advances in *Taxus* molecular research: applications in genetic diversity, gene function and metabolic engineering

**DOI:** 10.3389/fpls.2026.1880445

**Published:** 2026-08-10

**Authors:** Yan Jiang, Lingxiao Zhang, Huijie Ma, Zijin Fang, Chunna Yu, Xiaori Zhan, Yanjun Yang, Chenjia Shen

**Affiliations:** 1 College of Pharmacy, Hangzhou Normal University, Hangzhou, China; 2 College of Life and Environmental Sciences, Hangzhou Normal University, Hangzhou, China; 3 Research Center for Quality Evaluation of Dao-di Herbs, Nanchang, Jiangxi, China

**Keywords:** genetic diversity, molecular marker, multi-omics, omics, Taxol biosynthesis, *Taxus*

## Abstract

Plants belonging to the genus *Taxus* are abundant in pharmacologically active constituents, notably Taxol (paclitaxel), which has been extensively employed in the treatment of various cancers. This review aims to achieve a comprehensive and up-to-date overview of the roles of omics technologies in exploring genetic diversity, molecular identification, functional genes, and Taxol biosynthesis within the *Taxus* genus. Molecular markers derived from chloroplast, mitochondrial, and nuclear genomes, such as microsatellites, have been developed to assess genetic variability, spatial distribution, varietal differentiation, kinship structures, and fluctuations in inbreeding levels among *Taxus* populations. Functional gene identification guided by omics approaches has elucidated several critical genes implicated in the Taxol biosynthetic pathway, including *FoTO1*, *T1OH*, *T9αOH*, *T9α oxidase*, *C4β–C20 epoxidase*, and *Taxane Oxetanase 1* (*TOT1)*/*CYP725A55*/*TmCYP1*. Expression profiling aids in screening candidate genes related to Taxol biosynthesis, defense response, and growth and development process. Current advancements in omics technologies primarily focus on enhancing resolution, improving data visualization, and integrating multi-omics datasets. Looking forward, innovations such as telomere-to-telomere genome sequencing, spatial transcriptomics, and comparative genomics are anticipated to inaugurate a new epoch in omics research pertaining to *Taxus*. Our review aims to accelerate the efficient utilization of these endangered gymnosperms.

## 1 Introduction

**Figure 1:**
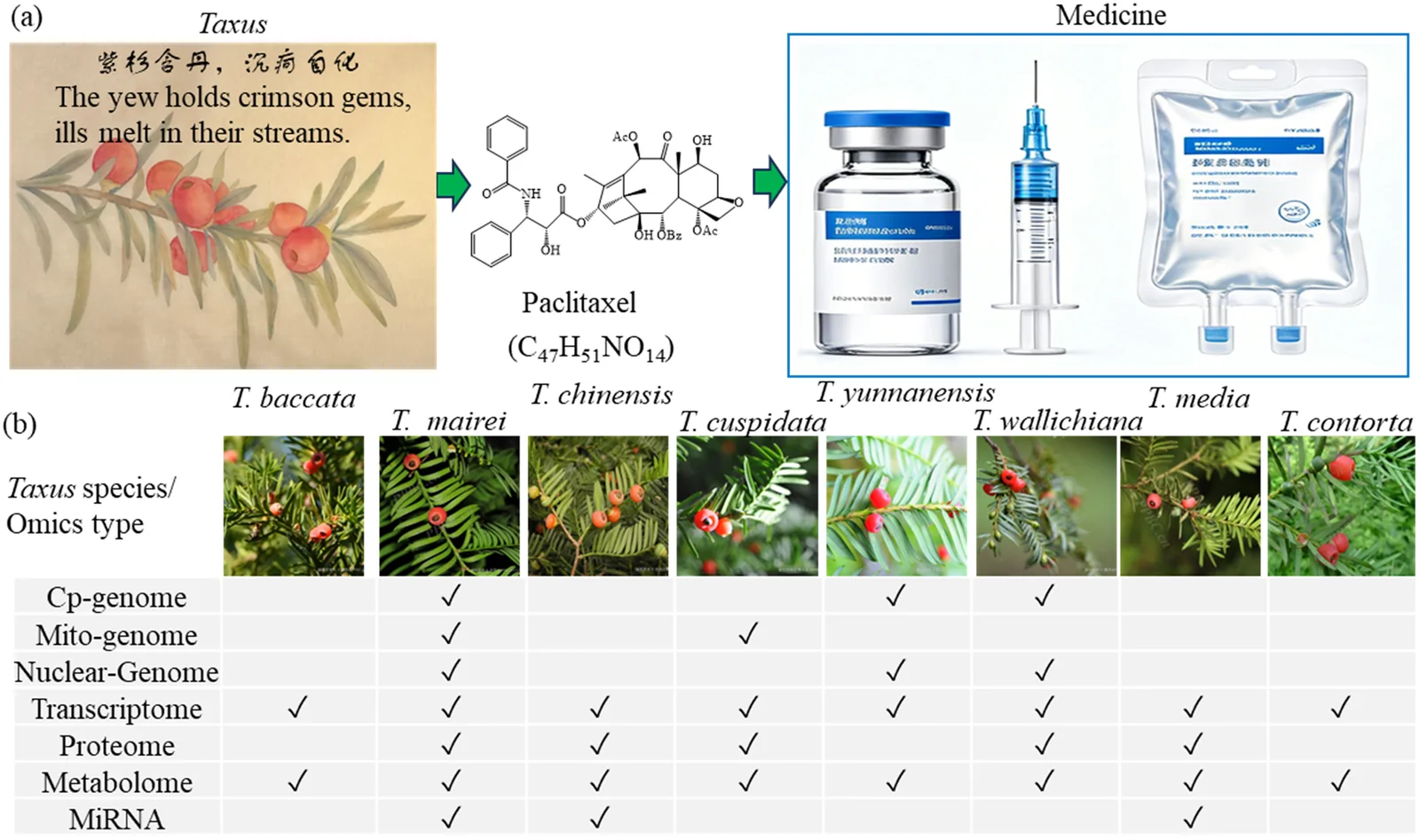
Overview of the omics researches on *Taxus* genus. **(a)** The active constituent of *Taxus* trees and its pharmacodynamic effects. **(b)** Summary of various omics data of *Taxus* genus. ‘✓’ indicated the availability of certain types of omics data. The pictures of various Taxus species were downloaded from Plant Photo Bank of China (https://ppbc.iplant.cn/).

*Taxus* L. belongs to the order Taxales and the family Taxaceae. It is an evergreen gymnosperm that lacks resin structure (Ghimire et al., 2014). As a relict plant from Quaternary glacier period, it is referred to a ‘living fossil’ of the plant kingdom (Mayol et al., 2015). These species are rich in pharmacologically active ingredients, including Taxol (generic name paclitaxel), flavonoids, and polysaccharides (Jiang et al., 2019; Zhou et al., 2019b). Taxol, an essential anticancer drug, was firstly extracted from the bark of *Taxus* species and has been broadly applied in the treatment of various cancers, such as breast, esophageal, lung, and stomach cancers (**Figure 1a**) (Yu et al., 2025b). The rising economic value of Taxol has led to extensive illegal logging, which combined with environmental degradation, threatens the survival of wild *Taxus* trees (Zhang et al., 2025). Fortunately, most wild *Taxus* species are now extensively cultivated for their ornamental and medicinal value in many countries.

Recently, there has been increasing attention on the genus *Taxus* in molecular research related to taxonomy, evolution, genetic diversity, secondary metabolism, environmental adaptation, and growth and development (Gao et al., 2024). Earlier reviews on *Taxus* species have largely focused on taxane chemistry and paclitaxel metabolic engineering, lacking a consolidated overview of omics tools bridging population genetics and biosynthetic gene discovery (Chen et al., 2025; Coombe-Tennant et al., 2025). Advances in technology have facilitated the widespread application of omics approaches in botanical studies. To date, massive omics data have been uploaded to NCBI BioProject database, including chloroplast genome, mitochondrial genome, nuclear genome, transcriptome, proteome, metabolome, and miRNAome (**Figure 1b**; **Table 1**). Here, we aim to give a full analysis of the use of omics in exploring genetic diversity, molecular identification, functional genes, and Taxol biosynthesis within the *Taxus* genus. Distinct from earlier overviews, the present review integrates population omics and functional multi-omics, highlights newly characterized enzymes and prospects emerging technologies such as telomere-to-telomere assembly and spatial transcriptomics for future *Taxus* research.

**Table 1:** The detailed information of omics data from NCBI.

	Species	Accession	Project data type	Date	Organization name	Reference
1	*T. mairei*	PRJNA79889	Transcriptome or Gene expression	20/12/2011	Dalian Jiaotong University	https://www.ncbi.nlm.nih.gov/bioproject/79889
2	*T. baccata*	PRJNA79759	Transcriptome or Gene expression	20/12/2011	JGI	https://www.ncbi.nlm.nih.gov/bioproject/79759
3	*T. cuspidata*	PRJNA79539	Transcriptome or Gene expression	20/12/2011	IMPLAD	https://www.ncbi.nlm.nih.gov/bioproject/79539
4	*T. media*	PRJNA169654	Transcriptome or Gene expression	30/06/2012	Chinese Academy of Forestry	https://www.ncbi.nlm.nih.gov/bioproject/169654
5	*T. mairei*	PRJNA170340	Transcriptome or Gene expression	11/07/2012	Murdoch University	https://www.ncbi.nlm.nih.gov/bioproject/170340
6	*T. chinensis*	PRJNA171206	Transcriptome or Gene expression	24/07/2012	Murdoch University	https://www.ncbi.nlm.nih.gov/bioproject/171206
7	*T. mairei*	PRJNA173133	Transcriptome or Gene expression	18/08/2012	Dalian Jiaotong University	https://www.ncbi.nlm.nih.gov/bioproject/173133
8	*T. cuspidata*	PRJEB2292	Transcriptome or Gene expression	21/05/2013	University of Edinburgh	https://www.ncbi.nlm.nih.gov/bioproject/203931
9	*T. chinensis*	PRJNA251671	Transcriptome or Gene expression	05/06/2014	Institute of Resource Biology and Biotechnology	https://www.ncbi.nlm.nih.gov/bioproject/251671
10	*T. wallichiana*	PRJNA294339	Raw sequence reads	31/08/2015	Sun Yat-Sen University	https://www.ncbi.nlm.nih.gov/bioproject/294339
11	*T. chinensis*	PRJNA360896	Transcriptome or Gene expression	11/01/2017	Central South University	https://www.ncbi.nlm.nih.gov/bioproject/360896
12	*T. florinii*	PRJNA384111	Raw sequence reads	25/04/2017	University of Chinese Academy of Sciences	https://www.ncbi.nlm.nih.gov/bioproject/384111
13	*T. baccata*	PRJNA393771	Transcriptome or Gene expression	11/07/2017	Institute of Applied Genomics	https://www.ncbi.nlm.nih.gov/bioproject/393771
14	*T. yunnanensis*	PRJNA427840	Raw sequence reads	28/12/2017	Sun Yat-sen University	https://www.ncbi.nlm.nih.gov/bioproject/427840
15	*T. media*	PRJNA436238	Raw sequence reads	28/02/2018	Tsinghua University	https://www.ncbi.nlm.nih.gov/bioproject/436238
16	*T. media*	PRJNA436829	Raw sequence reads	04/03/2018	Tsinghua University	https://www.ncbi.nlm.nih.gov/bioproject/436829
17	*T. media*	PRJNA436823	Raw sequence reads	04/03/2018	Tsinghua University	https://www.ncbi.nlm.nih.gov/bioproject/436823
18	*T. cuspidata*	PRJNA493167	Transcriptome or Gene expression	26/09/2018	Institute of Medicinal Plant Development	https://www.ncbi.nlm.nih.gov/bioproject/493167
19	*Taxus*	PRJNA497542	Transcriptome or Gene expression	19/10/2018	Hangzhou Normal University	https://www.ncbi.nlm.nih.gov/bioproject/497542
20	*Taxus*	PRJNA497542	Transcriptome or Gene expression	19/10/2018	Hangzhou Normal University	https://www.ncbi.nlm.nih.gov/bioproject/497542
21	*Taxus*	PRJNA497757	Raw sequence reads	22/10/2018	Institute of Resource Biology and Biotechnology	https://www.ncbi.nlm.nih.gov/bioproject/497757
22	*Taxus*	PRJNA498605	Transcriptome or Gene expression	26/10/2018	Hangzhou Normal University	https://www.ncbi.nlm.nih.gov/bioproject/498605
23	*T. contorta*	PRJNA499080	Raw sequence reads	29/10/2018	Central University of Punjab	https://www.ncbi.nlm.nih.gov/bioproject/499080
24	*T. mairei*	PRJNA524772	Transcriptome or Gene expression	28/02/2019	Jiangsu Province and Chinese Academy of Sciences	https://www.ncbi.nlm.nih.gov/bioproject/524772
25	*T. cuspidata*	PRJNA553347	Chloroplast genome	09/07/2019	Beijing Forestry University	https://www.ncbi.nlm.nih.gov/bioproject/553347
26	*T. fuana*	PRJNA554197	Chloroplast genome	11/07/2019	Beijing Forestry University	https://www.ncbi.nlm.nih.gov/bioproject/554197
27	*T. wallichiana*	PRJNA554072	Chloroplast genome	11/07/2019	Beijing Forestry University	https://www.ncbi.nlm.nih.gov/bioproject/554072
28	*Gymnosperms*	PRJNA578185	Mitochondrial genome	17/10/2019	Chinese Academy of Sciences	https://www.ncbi.nlm.nih.gov/bioproject/578185
29	*T. chinensis*	PRJNA580323	Transcriptome or Gene expression	19/10/2019	Chinese Academy of Forestry	https://www.ncbi.nlm.nih.gov/bioproject/580323
30	*T. media*	PRJNA586725	Raw sequence reads	31/10/2019	Jiangsu Province and Chinese Academy of Sciences	https://www.ncbi.nlm.nih.gov/bioproject/586725
31	*T. media*	PRJNA651763	Transcriptome or Gene expression	04/08/2020	DOE Joint Genome Institute	https://www.ncbi.nlm.nih.gov/bioproject/651763
32	*T. yunnanensis*	PRJNA661543	Genome sequencing and assembly	11/05/2021	China Academy of Chinese Medical Sciencess	https://www.ncbi.nlm.nih.gov/bioproject/661543
33	*T. media*	PRJNA733140	Transcriptome or Gene expression	27/05/2021	Hangzhou Normal University	https://www.ncbi.nlm.nih.gov/bioproject/733140
34	*T. media*	PRJNA733140	Transcriptome or Gene expression	27/05/2021	Hangzhou Normal University	https://www.ncbi.nlm.nih.gov/bioproject/733140
35	*T. chinensis*	PRJNA735009	Genome sequencing	03/06/2021	Chinese academy of agriculture sciences	https://www.ncbi.nlm.nih.gov/bioproject/735009
36	*T. chinensis*	PRJNA751266	Raw sequence reads	01/08/2021	Jiangsu Normal University	https://www.ncbi.nlm.nih.gov/bioproject/751266
37	*T. chinensis*	PRJNA730337	Genome sequencing and assembly	26/08/2021	Chinese academy of agriculture sciences	https://www.ncbi.nlm.nih.gov/bioproject/730337
38	*T. chinensis*	PRJNA789168	Transcriptome or Gene expression	15/12/2021	Jiangsu Normal University	https://www.ncbi.nlm.nih.gov/bioproject/789168
39	*T. baccata*	PRJNA791291	Raw sequence reads	21/12/2021	IGA Technology Services Srl	https://www.ncbi.nlm.nih.gov/bioproject/791291
40	*T. cuspidata*	PRJNA797697	Transcriptome or Gene expression	16/01/2022	Northeast Forestry University	https://www.ncbi.nlm.nih.gov/bioproject/797697
41	*T. cuspidata*	PRJNA839234	Transcriptome or Gene expression	18/05/2022	Worcester Polytechnic Institute	https://www.ncbi.nlm.nih.gov/bioproject/839234
42	*T. chinensis*	PRJNA848951	Raw sequence reads	14/06/2022	Huazhong Agricultural University	https://www.ncbi.nlm.nih.gov/bioproject/848951
43	*T. mairei*	PRJNA858957	Raw sequence reads	15/07/2022	Tianjin University of Science and Technology	https://www.ncbi.nlm.nih.gov/bioproject/858957
44	*Taxus*	PRJNA863389	Genome sequencing	28/07/2022	University of Chinese Academy of Sciences	https://www.ncbi.nlm.nih.gov/bioproject/863389
45	*Taxus*	PRJNA864083	Transcriptome or Gene expression	31/07/2022	University of Chinese Academy of Sciences	https://www.ncbi.nlm.nih.gov/bioproject/864083
46	*Taxus*	PRJNA864114	Raw sequence reads	01/08/2022	University of Chinese Academy of Sciences	https://www.ncbi.nlm.nih.gov/bioproject/864114
47	*T. media*	PRJNA900696	Raw sequence reads	12/11/2022	Tianjin University of Science and Technology	https://www.ncbi.nlm.nih.gov/bioproject/900696
48	*T. mairei*	PRJNA904824	Raw sequence reads	23/11/2022	Tianjin University of Science and Technology	https://www.ncbi.nlm.nih.gov/bioproject/904824
49	*T. mairei*	PRJNA909435	Raw sequence reads	07/12/2022	Hangzhou Normal University	https://www.ncbi.nlm.nih.gov/bioproject/909435
50	*T. media*	PRJDB15908	Transcriptome or Gene expression	17/05/2023	Kyoto University	https://www.ncbi.nlm.nih.gov/bioproject/973866
51	*T. yunnanensis*	PRJNA980435	Raw sequence reads	06/06/2023	Jiangsu normal university	https://www.ncbi.nlm.nih.gov/bioproject/980435
52	*T. mairei*	PRJNA980442	Transcriptome or Gene expression	06/06/2023	Minzu University of China	https://www.ncbi.nlm.nih.gov/bioproject/980442
53	*T. yunnanensis*	PRJNA991749	Raw sequence reads	06/07/2023	Yunnan Academy of Forestry and Grassland	https://www.ncbi.nlm.nih.gov/bioproject/991749
54	*T. media*	PRJNA999506	Raw sequence reads	28/07/2023	Chinese Academy of Agricultural Sciences	https://www.ncbi.nlm.nih.gov/bioproject/999506
55	*T. chinensis*	PRJNA1002123	Raw sequence reads	04/08/2023	Xinyang Agriculture and Forestry University	https://www.ncbi.nlm.nih.gov/bioproject/1002123
56	*T. mairei*	PRJNA1003858	Transcriptome or Gene expression	09/08/2023	Central South University of Forestry and Technology	https://www.ncbi.nlm.nih.gov/bioproject/1003858
57	*T. cuspidata*	PRJDB16626	Transcriptome or Gene expression	15/09/2023	Hokkaido University	https://www.ncbi.nlm.nih.gov/bioproject/1017995
58	*T. mairei*	PRJNA1023917	Raw sequence reads	04/10/2023	Hangzhou Normal University	https://www.ncbi.nlm.nih.gov/bioproject/1023917
59	*T. mairei*	PRJNA1025358	Raw sequence reads	08/10/2023	Hangzhou Normal University	
60	*T. chinensis*	PRJNA1031429	Raw sequence reads	24/10/2023	Nanjing Forestry University	https://www.ncbi.nlm.nih.gov/bioproject/1031429
61	*Taxus*	PRJNA1062083	Raw sequence reads	07/01/2024	The National Omics Data Encyclopedia	https://www.ncbi.nlm.nih.gov/bioproject/1062083
62	*T. wallichiana*	PRJNA1068470	Raw sequence reads	24/01/2024	Chinese Academy of Sciences	https://www.ncbi.nlm.nih.gov/bioproject/1068470
63	*Taxus*	PRJNA1073191	Raw sequence reads	04/02/2024	Hangzhou Normal University	https://www.ncbi.nlm.nih.gov/bioproject/1073191
64	*T. mairei*	PRJNA1096280	Transcriptome or Gene expression	04/04/2024	Hangzhou Normal University	https://www.ncbi.nlm.nih.gov/bioproject/1096280
65	*T. chinensis*	PRJNA1097243	Raw sequence reads	07/04/2024	Fujian Academy of Forestry Sciences	https://www.ncbi.nlm.nih.gov/bioproject/1097243
66	*T. wallichiana*	PRJNA1132188	Raw sequence reads	05/07/2024	Shandong Provincial Center of Forest and Grass Germplasm Resources	https://www.ncbi.nlm.nih.gov/bioproject/1132188
67	*T. mairei*	PRJNA1140359	Raw sequence reads	25/07/2024	South China University of Technology	https://www.ncbi.nlm.nih.gov/bioproject/1140359
68	*T. media*	PRJNA1241716	Transcriptome or Gene expression	19/05/2025	Stanford University	https://www.ncbi.nlm.nih.gov/bioproject/1241716
69	*T. cuspidata*	PRJNA1306783	Transcriptome or Gene expression	16/08/2025	Changchun Normal University	https://www.ncbi.nlm.nih.gov/bioproject/1306783
70	*T. mairei*	PRJNA909435	Single-cell datasets	11/09/2023	Hangzhou Normal University	https://www.ncbi.nlm.nih.gov/bioproject/909435
71	*T. mairei*	PRJNA730337	Single-cell datasets	01/09/2023	Hangzhou Normal University	https://www.ncbi.nlm.nih.gov/bioproject/730337

Despite continuous technological breakthroughs in omics investigation of *Taxus*, several fundamental research gaps remain underexplored. The regulatory networks coordinating taxane biosynthesis, stress adaptation, and the mechanisms underlying resource trade-offs between primary metabolism and paclitaxel accumulation remain poorly characterized. Prioritized future research should focus on the bridging basic omics data with practical conservation and utilization of wild yew resources.

## 2 Omics technologies facilitate the development of molecular markers

**Figure 2:**
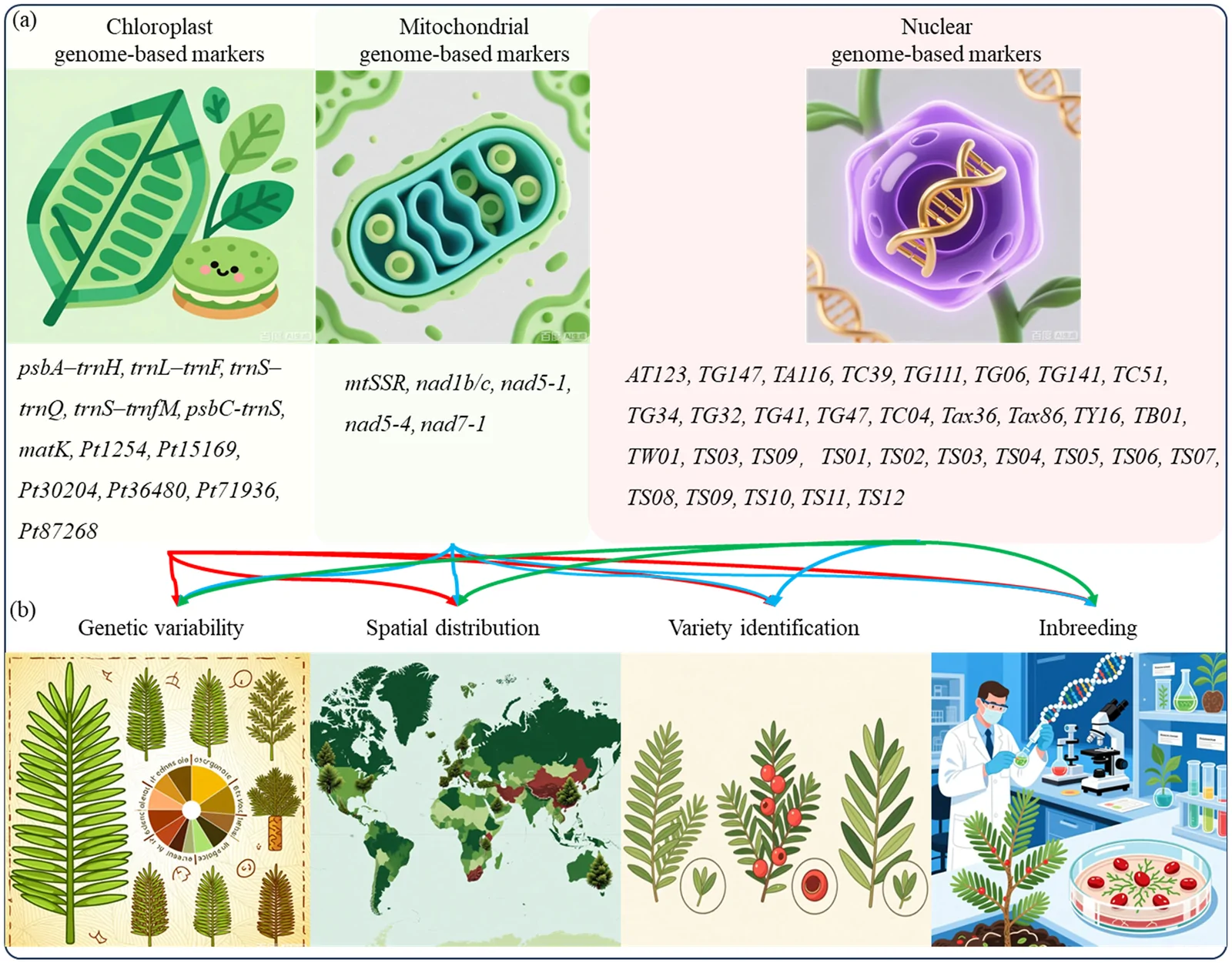
Omics technologies facilitate the development of molecular markers of *Taxus*. **(a)** A detailed list of molecular markers derived from chloroplast, mitochondrial, and nuclear genomes. **(b)** Application of molecular markers in the analysis of genetic variability, spatial distribution, variety identification, and inbreeding.

Phenotypic, ecological, and genetic differences among closely related species are crucial for understanding speciation processes (Nieto Feliner, 2014). Species are the fundamental units of biodiversity, but distinguishing between similar species remains a challenging task (Wu et al., 2024). *Taxus* is the genus with the largest number of species within the Taxaceae family. According to a recent study by Möller et al., the genus *Taxus* comprises 16 genetically defined lineages, including *T. baccata*, *T. brevifolia*, *T. calcicola*, *T. canadensis*, *T. chinensis*, *T. contorta*, *T. cuspidata*, *T. floridana*, *T. florinii*, *T. globosa*, *T. mairei*, *T. phytonii*, *T. emei* type, *T. qinlingensis*, and *T. huangshanensis*, which are primarily distributed across the Northern Hemisphere (Möller et al., 2020). Due to phenotypic plasticity, the classification of *Taxus* still is controversial (Gao et al., 2007). DNA markers are frequently used to distinguish differences between plant species (Hao et al., 2018). Here, we provide a detailed review of molecular markers applicable for the identification of *Taxus* species (**Figure 2a**).

### 2.1 Chloroplast genome-based markers

Chloroplast genomes are valuable for phylogenetic analysis and species identification because they possess a relatively conserved structure and exhibit a low mutation rate (Palmer et al., 1988). Determining the chloroplast genome contributes to species identification, phylogenetic analysis, genetic diversity evaluation, adaptive evolution research, germplasm resource utilization, and conservation biology of plants. To date, the complete chloroplast genomes of four *Taxus* species, such as *T. yunnanensis* (129, 190 bp and 116 gene copies), *T. wallichiana* (128, 168 bp and 116 gene copies), and *T. mairei* (129, 513 bp and 113 gene copies), have been published (Zhang et al., 2014; Geng et al., 2020; Ruan et al., 2020). Additionally, the chloroplast genome of *Amentotaxus argotaenia*, a sister genus to *Taxus*, also has been determined (Li et al., 2016). Comparative analysis of chloroplast genomes revealed that they are highly conserved, offering a range of potential conserved sites for developing molecular markers in the *Taxus* genus (Liu et al., 2019).

Nucleotide polymorphism of intergenic spacers, including *psbA*–*H*, *trnL–F*, *trnS–Q*, and *trnS–M*, in chloroplast genome were used for the study on the genetic diversity of *Taxus* populations (Schirone et al., 2010; Kozyrenko et al., 2017). Eighteen chloroplast-based microsatellites were developed from *T*. *mairei* and successfully applied in the populations of other Taxaceae genus, such as *Pseudotaxus chienii* (Deng et al., 2017). Based on two chloroplast DNA sequences, *trnL-F* and *psbC-trnS*, the genetic variation of *T. sumatrana* in Indonesia was determined (Rachmat et al., 2016). Poudel et al., reconstructed the genetic diversity and demographic history of *T. contorta* in Pakistan by analyzing chloroplast DNA sequence (*trnL*-*F*) (Poudel et al., 2014). Eight chloroplast DNA markers, including *Pt1254*, *Pt15169*, *Pt30204*, *Pt36480*, *Pt71936*, and *Pt87268*, were used for the divergence of *T. baccata* populations across the Strait of Gibraltar (Jaramillo-Correa et al., 2010). The chloroplast *matK* gene has be used to study the phylogeny of Taxaceae genera (Cheng et al., 2000). The chloroplast *trn*L-F intron-spacer region was used to study the phylogeographic patterns of *T. wallichiana* in China and northern Vietnam (Gao et al., 2007).

### 2.2 Mitochondrial genome-based markers

Thousands of chloroplast genomes have been sequenced, but only a few hundred mitochondrial genomes have been characterized, due to the remarkable variability in both structure and sequence content of plant mitogenomes (Štorchová and Krüger, 2024). To date, the mitochondrial genomes of two *Taxus* species, such as *T. wallichiana* (469, 949 bp and 42 genes) and *T. cuspidata* (468, 924 bp and 46 genes), have been published (Kan et al., 2020; Qu et al., 2024). Considering that chloroplast genomes provide insufficient genetic information to differentiate closely related plant species, mitogenomes are reported to contain more phylogenetic signals (Lloyd Evans et al., 2019).

Several mitochondrial simple sequence repeat markers have been developed to examine the genetic patterns of *T. mairei* populations (Wen et al., 2018). Additionally, two markers based on mitochondrial DNA variants were created to reveal the genetic structures of *T. cuspidata* in Changbai Mountain, in Northeast China (Su et al., 2018). Furthermore, four mitochondrial markers, such as *nad1b/c*, *nad5-1*, *nad5–4* and *nad7-1*, were also used in *T. baccata* survey (Jaramillo-Correa et al., 2010). In the future, mitochondrial molecular resources provide reliable genetic data for comparative evolutionary analysis of gymnosperms, help clarify the evolutionary divergence and adaptive differentiation pattern of gymnosperm lineages, and lay a foundation for the conservation genetics and germplasm resource evaluation of rare and endangered gymnosperm plants.

### 2.3 Nuclear genome-based microsatellite markers

Microsatellite markers, also known as simple sequence repeats (SSRs), are simple repeat sequences uniformly distributed throughout plant genomes, consisting of tandem repeats of 2–6 nucleotides (Hokanson et al., 1998). SSR markers have been employed for genotyping populations using gel electrophoresis and combined with staining (Huang et al., 2018). In the past years, numerous microsatellite markers have been developed and applied to various *Taxus* species, including *T. baccata*, *T. contorta*, *T. chinensis*, *T. wallichiana*, *T. mairei*, *T. florinii* and *T. cuspidata* (Fu et al., 2013). Here, we summarize the sources and applications of SSR in *Taxus* genus.

In the early days, most SSR markers for *Taxus* were identified using an enriched-library method (Edwards et al., 1996). Polymorphism loci of DNA containing di-(GT, AT) and trinucleotide (GCC) repeats were sequenced to screen highly polymorphic SSRs that were used for population genetic analysis (Dubreuil et al., 2008). For examples, seven nuclear microsatellite markers in *T. baccata*, eight SSR loci and eleven microsatellite loci in *T. mairei*, ten microsatellite markers in *T. wallichiana*, twelve microsatellite loci in *T. sumatrana*, and eleven polymorphic microsatellites in *T. yunnanensis* have been developed using this traditional method (Huang et al., 2008; González-Martínez et al., 2010; Xue et al., 2012; Zhang and Zhou, 2013; Miao et al., 2014). Furthermore, Modified double-digest restriction-site-associated DNA sequencing (MiddRAD-seq) data can also be used to identify microsatellite markers of endangered *T. florinii* (Qin et al., 2017).

Recently, thousands of plant Expressed Sequence Tags (ESTs) containing SSR loci have been detected using high-throughput transcriptome sequencing. For instance, transcriptomic analyses of *T. contorta*, *T. baccata*, and *T. cuspidata* predicted 7, 041, 2, 330, and 7, 404 SSR markers, respectively, thereby expanding the sources of SSR markers (Ueno et al., 2015; Olsson et al., 2018; Majeed et al., 2019). Collectively, microsatellite markers in *Taxus* have been progressively developed through traditional enriched-library approaches, MiddRAD-seq, and high-throughput transcriptome sequencing, providing abundant molecular resources for population genetics and evolutionary studies of the genus.

### 2.4 Application of molecular markers in the evaluation of genetic diversity

Due to geographical isolation, ancient populations of *Taxus* frequently exhibit a clearly disjunct distribution (Spanu et al., 2015). The evaluation of genetic diversity requires appropriate and reliable genetic markers. Huang’s group employed SSR markers to investigate the level of genetic variability between populations of *T. chinensis* and *T. wallichiana* in Vietnam (Vu et al., 2017). Using microsatellite markers, Bouza’s group examined the spatial distribution of forty-six populations of *T. baccata* in northern Spain (Maroso et al., 2021). Due to climatic differences and artificial cultivation, a species of *Taxus* often has multiple variants. In 2016, Toshiaki Kondo developed 15 microsatellite markers to distinguish the dwarf *T. cuspidata* var. *nana* variety from common *T. cuspidata* in Japan (Kondo, 2016). Recently, both chloroplast DNA and nuclear SSR revealed that genetic diversity of *T. fuana* in Tibet, China, declined in a size-dependent manner (Shen-Tu et al., 2025).

### 2.5 Application of molecular markers in conservation

Additionally, SSR genotyping has be used to identify genetic diversity hotspots, which plays important roles in conservation management for the Himalayan endangered species, *T. contorta* (Majeed et al., 2021). Microsatellite markers can also be used for calculating the estimated kinship structure and conservation of *T. baccata* populations (Chybicki et al., 2011). Ten microsatellite markers were developed and used for further investigations of the conservation genetics of the endangered species *T. wallichiana* (Liu et al., 2011). Another ten highly polymorphic microsatellite markers were applied to the conservation of the endangered tree species *T. wallichiana* from Nepal (Gajurel et al., 2013).

In summary, microsatellite markers are powerful tools for analyzing genetic variability, spatial distribution, varietal variation, kinship structure, and shifts in inbreeding levels within *Taxus* populations (**Figure 2b**).

### 2.6 Historical progression of genetic markers applied in genus *Taxus*

Genetic investigation of *Taxus* has undergone four successive technical phases. In 1990s, allozyme electrophoresis quantified population variation and mating systems of *T. baccata* and *T. cuspidata*, yet limited polymorphic loci restricted its wide use on rare endemics (Lewandowski et al., 1992; Chung et al., 1999). From the late 1990s to early 2000s, RAPD and ISSR replaced allozymes for rapid species identification, though poor repeatability limited cross-lab comparisons (Zarek, 2009). Codominant markers (SSRs, cpDNA, mtDNA) then dominated, resolving interspecific boundaries and genetic erosion for conservation genetics, and remain widely used today.

## 3 Omics researches on *Taxus* genes

### 3.1 Genes involved in Taxol biosynthesis

Taxol is one of the most valuable chemotherapeutic agents and is broadly applied in the treatment of different cancers, including ovarian cancer, breast cancer and squamous cancer (Shao et al., 2021). To obtain sufficient quantities of Taxol, several biological synthesis strategies have been developed based on the known steps of the Taxol biosynthesis pathway (Shi et al., 2025). Thus, functional identification of genes involved in Taxol biosynthesis is essential for developing efficient heterologous synthesis systems. Genes related to Taxol biosynthesis can be categorized into two groups: those encoding enzymes involved in Taxol biosynthesis and those encoding regulatory proteins (Yu et al., 2021; Coombe-Tennant et al., 2025). We explore the use of omics technologies to identify genes involved in Taxol biosynthesis from these two perspectives.

Before the widespread application of omics technologies, several significant methods, such as feeding experiments, cell-free enzymology, library construction, and functional gene cloning, were employed to reveal gene functions (Croteau et al., 2006; Coombe-Tennant et al., 2025). The intricate biosynthetic pathway of Taxol involves approximately 20 steps from the diterpenoid taxane core to the final product with a C13-side chain (Eisenreich et al., 1996). However, molecular evidence of several critical steps in the formation of certain intermediates, such hypothetical reactions that presumably catalyzed by phenylalanine-CoA ligase (PCL), taxane 1b-hydroxylase (T1bOH), taxane 9a-hydroxylase (T9aOH), and taxane 9a-dioxygenase, remain unknown (Li et al., 2019; Zhang et al., 2023). With the advancements in omics technologies, analyzing these unknown steps has gradually become feasible. Prior to the advent of omics, the expression profiles of the Taxol biosynthetic pathway genes were analyzed using gel blot and/or qRT-PCR methods (Nims et al., 2006; Brunáková and Kosuth, 2009; Li et al., 2009).

#### 3.1.1 Transcriptome-guided functional gene identification

**Figure 3:**
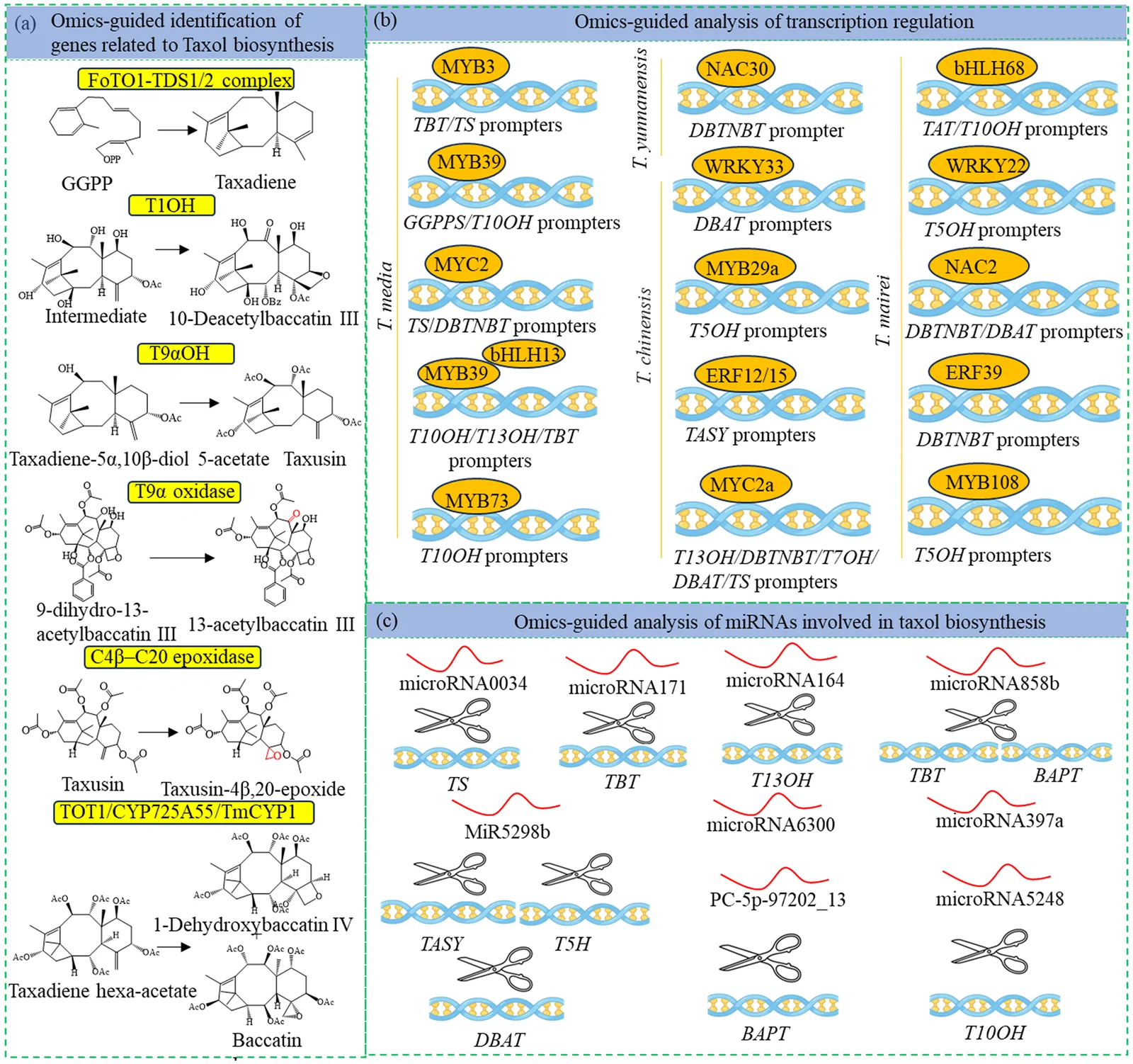
Omics researches on Taxol biosynthesis of *Taxus*. **(a)** Omics-guided identification of genes-related to Taxol biosynthesis. **(b)** Omics-guided analysis of transcription regulation. **(c)** Omics-guided analysis of miRNAs involved in Taxol biosynthesis.

Subsequently, transcriptomic technology was widely used in *Taxus* research. There are seven published whole transcriptome datasets: *T. mairei*, *T. yunnanensis*, *T. cuspidata*, *T. media*, *T. baccata*, *T. chinensis*, and *T. wallichiana*. A 454 pyrosequencing technology was utilized to generate a transcriptome of *T. cuspidata* needles, identifying 14, 095 unigenes (Wu et al., 2011). In the same year, a *de novo* assembled transcriptome of *T. mairei* produced 23, 515 unigenes (Hao da et al., 2011). The first transcriptomes of *T. chinensis* (Li et al., 2012), *T. media* (Sun et al., 2013), *T. baccata* (Olsson et al., 2018), *T. yunnanensis* (He et al., 2018), *T. wallichiana* (Wang et al., 2019), and *T. contorta* (Majeed et al., 2020), were also published. Based on similarity searches with known genes, most of the Taxol biosynthesis genes can be detected through transcriptome sequencing. Iso-Seq analysis of *T. cuspidata* identified nine CYP450 genes and seven BAHD ACT genes as potential key candidates for Taxol biosynthesis (Kuang et al., 2019). *In silico* mining of several publicly available transcriptomic datasets revealed a full list of CYP450 superfamily members, providing a sufficient way for identifying the missing bottleneck enzyme-encoding genes (Liao et al., 2017). Using the transcriptomic analysis, Fernie’s group identified two previously unknown hydroxylase genes (*T9αOH*, *T1βOH*) and one previously unknown oxidase gene (*T9α oxidase*), which are considered to be involved in the baccatin III biosynthesis pathway (Zhang et al., 2023). Furthermore, the same group predicted a C4β-C20 epoxidase encoding gene (Zhang et al., 2023). After analyzing genomic and transcriptomic datasets, Kampranis’s group revealed that CYP725A4 catalyzes two continuous epoxidation steps, promoting to the formation of oxetane ring oxetane (Zhao et al., 2024). Searching a transcriptome database of paclitaxel-producing *T. media* cell cultures, 22 full-length highly expressed *CYP725A* transcripts (*TmCYP1*–*TmCYP22*) were obtained, assisting in the identification of oxetane synthase (TmCYP1), a novel enzyme catalyzing the formation of the oxetane ring in tetracyclic taxoids (Li et al., 2024). Recently, FoTO1 was reported to participate in the initial oxidation of baccatin III biosynthesis using a multiplexed perturbation × single nuclei transcriptomic method (**Figure 3a**) (McClune et al., 2025).

#### 3.1.2 Nuclear genome-guided functional gene identification

With the advent of the genomic era, an increasing number of missing genes have been consecutively cloned and functionally validated. Three chromosome-level genomes of different *Taxus* species, including *T. mairei* (10.23 Gb), *T. yunnanensis* (10.7 Gb), *T. wallichiana* (10.9 Gb), have been published (Cheng et al., 2021; Song et al., 2021; Xiong et al., 2021). Recently, phased high-quality genomes of two *T. wallichiana* haplotypes (9.87 Gb and 9.98 Gb) were assembled, each chromosome with telomeres at both ends (Li et al., 2025b). Based on the *T. wallichiana* genome, a batch of 2-oxoglutarate/Fe(II)-dependent dioxygenases, which were predicted to be key C4β–C20 epoxidases in the Taxol biosynthesis pathway, were identified (Li et al., 2025b). Based on the *T. mairei* genome, a research group identified that TOT1 catalyzes an oxidative rearrangement in Taxol oxetane formation (Jiang et al., 2024). Besides, another group identified a total of 123 BAHD acyltransferases, including several BAHD genes in the *Taxus*-specific branch based on the *T. mairei* genome (Xu et al., 2024).

Scientists have selected different species of *Taxus* as research materials, s leading to similar studies being conducted simultaneously across various *Taxus* species and resulting in a duplication of scientific efforts. Sequence analysis has revealed that CYP1 in *T. media*, TOT1 in *T. mairei*, and CYP725A55 in *T. yunnanensis* are homologous proteins that perform similar biological functions in catalyzing oxetane ester formation (Li et al., 2025a). Therefore, it is crucial to integrate omics data comprehensively, particularly by unifying gene identifiers. From transcriptome to genome, the biosynthetic pathway of Taxol in *Taxus* is expected to be fully elucidated in the near future (Fernie et al., 2024).

Despite accumulating genomic resources for the genus *Taxus*, several critical research gaps still restrict the comprehensive understanding of yew evolution and conservation genetics. First, most endemic narrow-range *Taxus* species lack chromosome-level nuclear genomes due to conservation restrictions and high sequencing costs. Second, pan-genomic resources of *Taxus* have not been established. Third, the genetic basis of sex differentiation and adaptive evolution under habitat fragmentation in wild yew populations remains poorly resolved. Last, large-scale population genomic data across natural distribution zones are lacking to formulate precise conservation strategies for endangered germplasm. It is believed that all the above problems can be solved in the near future.

#### 3.1.3 Expression profiling assists in screening candidate genes for Taxol synthesis

Although omics studies have identified some key enzyme-coding genes involved in paclitaxel synthesis, cell-type or tissue specific expression analysis remains the most effective approach for leveraging omics data. Given the bark-specific accumulation of taxoids, analyzing tissue-specific expression patterns provides a streamlined method for screening key genes related to Taxol biosynthesis (Uniyal, 2013; Bai and Li, 2024). The tissue-specific patterns of Taxol biosynthesis-related genes in *T. mairei* and *T. yunnanensis* were investigated (Hao da et al., 2011; He et al., 2018; Mubeen et al., 2018; Jiang et al., 2025). Co-expression network analysis of gene expression and taxoid accumulation predicted several Taxol biosynthesis pathway-related genes, such as *T2OH*, *TDAT*, and *T10OH*, which represent promising targets for artificial regulation (Mubeen et al., 2018; Jiang et al., 2025). A tissue-specific proteomic study identified three phloem-specifically expressed proteins, including TBT, DBTNBT and TS, explaining the phloem-specific accumulation of Taxol (Yu et al., 2020). To identify the potential T9aH enzyme responsible for C9 hydroxylation of the taxoid skeleton, 17 *CYP725A* candidates were screened by analyzing tissue-specific expression patterns, significantly narrowing the pool of candidate genes (Jiang et al., 2024).

In addition, omics technologies are powerful tools for analyzing interspecies differences in *Taxus* (Yu et al., 2021a). Comparative transcriptome and chloroplast genome analyses of two *T. yunnanensis* individuals identified several key genes, such as *T10βH*, *DBAT*, and *DBTNBT*, associated with high Taxol yield (Wang et al., 2024a). Comparative transcriptome analysis of three *Taxus* species revealed that the MEP pathway-associated genes, including *DXS*, *DXR*, *MCT*, *CMK*, *MDS*, *HDS*, *HDR*, *IPPI*, and *GGPPS*, play important roles in variations in taxoid content (Zhou et al., 2019a). Another comparative transcriptome analysis of three experimental lines of *T. yunnanensis* elucidated the regulatory mechanism underlying the biosynthesis of taxanes (Mubeen et al., 2018). Proteomic analysis of different *Taxus* species predicted three cytochrome P450 taxoid oxygenases involved in the hydroxylation steps of Taxol biosynthesis (Hao et al., 2017).

Activating the plant defense system using elicitors is an effective way to promote the accumulation of secondary metabolites (Zhan et al., 2022; Perez-Matas et al., 2023a). To date, various elicitors, such as cyclodextrin, salicylic acid, nitric oxide, Ce^4+^, endophytic fungus, light condition, perfluorodecalins, hexenol, methyl jasmonate (MeJA), and coronatine, have been used to enhance the accumulation of taxoids in *Taxus* plants (Wang and Wu, 2004; Onrubia et al., 2013; Vidal-Limon et al., 2018; Han et al., 2022; Li et al., 2022; Zhao et al., 2023). Among above elicitors, MeJA has been successfully and frequently applied in the enhancement of Taxol accumulation in various *Taxus* cultures (Yukimune et al., 1996). However, the regulation mechanisms underlying the MeJA-mediated taxoid biosynthesis is largely unknown. Transcriptomic analysis of *T. media* cells after MeJA treatments showed that MeJA-induced Taxol biosynthesis is a rapid response event (Mao et al., 2018). A genome-wide expression analysis of MeJA-elicited *T. baccata* cell cultures revealed that β-phenylalanine-CoA ligase is responsible for converting β-phenylalanine to target metabolite (Ramírez-Estrada et al., 2016). In Yan’s study (2024), MeJA was used to screen candidate genes by transcriptomic analysis (Jiang et al., 2024).

#### 3.1.4 Genes encoding transcription factorsinvolved in Taxol biosynthesis

Omics technologies have played a crucial role in identifying TFs involved in Taxol biosynthesis (Cai et al., 2025). A minimal transcription factor network can activate a subset of paclitaxel biosynthetic genes in stable transgenic *Taxus* cambial meristematic cell system (Pan et al., 2026). Based on omics data, the R2R3-MYB, AP2/EREBP, MYC, WRKY, and JAZ TF families in *Taxus* were identified (Zhang et al., 2017; Yanfang et al., 2018; Zhang et al., 2018, 2019; Hu et al., 2020). Based on the *T. yunnanensis* genome, 44 NAC genes were identified, among which the MeJA responsive *TyuNAC30*, which encodes a negative regulator of *DBTNBT*, a key gene in Taxol biosynthesis (Fan et al., 2025). Transcriptomic changes in *Taxus* induced by various elicitors led to the identification of several MeJA-responsive TF genes (*TcMYC* and *TcJAMYC5*), SA-responsive TF genes (*TcWRKY33*, *TcMYB73*, and *TcTGA6*), ABA-responsive TF gene (*TbMYB29a*), and JA-responsive TF genes (*TcERF12*, *TcERF15*, *TcJAMYC1*, *TcJAMYC2*, and *TcJAMYC4*) (Lenka et al., 2015; Zhang et al., 2015; Yanfang et al., 2018; Chen et al., 2021; Cao et al., 2022; Ying et al., 2023; Cai et al., 2025; Ren et al., 2025). These TF genes may be involved in elicitor-mediated enhancement of Taxol biosynthesis. In *T. media*, proteomic analysis of different stem tissues identified a phloem-specific TF, TmMYB3, that controls Taxol biosynthesis by activating the expression of *TBT* and *TS* genes (Yu et al., 2020). Transcriptomic analysis of *T. media* identified a female-specific R2R3-MYB TF gene, *TmMYB39*, which function in the transcriptional regulation of Taxol biosynthesis by transactivating the *GGPPS* and *T10OH* genes (Yu et al., 2022). Integrated untargeted metabolomic and transcriptomic analysis identified a fungal elicitor-induced TF, MYC2, which activated Taxol biosynthesis by regulating the expression of *TS*, and *DBTNBT* (Wang et al., 2025a). In *T. mairei*, single-cell transcriptomic analysis identified six leaf cell-specific TF genes (*MYB17*, *WRKY12*, *WRKY31*, *ERF13*, *GT_2*, and *bHLH46*) and three stem cell-specific TF genes (*MYB47*, *NAC2*, and *bHLH68*), revealing cell-specific regulation of Taxol biosynthesis (Yu et al., 2023; Zhan et al., 2023a). Single-cell transposase-accessible chromatin (ATAC) sequencing identified four additional leaf cell-specific TF genes (*bHLH18*, *MYB108*, *WRKY22*, and *ERF39*), revealing the roles of chromatin accessibility in regulating Taxol biosynthesis (Zhan et al., 2023b). Recently, transcriptomic analysis of *T. yunnanensis* and *T. media* revealed the key role of *MYB23* in regulating wax synthesis (Fan et al., 2026). With advances in genetic transformation systems, cloned TFs are considered to play an important role in the genetic improvement of *Taxus* (**Figure 3b**).

#### 3.1.5 MicroRNAs involved in Taxol biosynthesis

MiRNAs are essential regulators of gene expression involved in various secondary metabolic processes in *Taxus* trees (Sabzehzari and Naghavi, 2019; Sun et al., 2024). In *T. chinensis*, high-throughput Illumina sequencing identified 58 miRNAs grouping into 25 subfamilies (Qiu et al., 2009). High-throughput degradome sequencing detected 871 mature miRNAs, 15 miRNA and 869 miRNA precursors in *T. mairei* (Hao et al., 2012). A deep sequencing approach provided a number of target miRNAs that involved in Taxol biosynthesis. For an example, a novel miRNA, *m0034*, was predicted to be a regulator of gene *TS*, while *T13OH* and *TBT* were identified as the targets of *miR164* and *miR171*, respectively (Hao et al., 2012). In *T. media*, *T10OH* was controlled by *miR5248* and *miR397a*, and *BAPT* was regulated by *gma-miR6300* and *PC-5p-97202_13* (Chen et al., 2020). *MiR5298b* promoted the biosynthesis of Taxol and upregulated the expression of several Taxol biosynthesis-related genes, including *DBAT*, *TASY*, and *T5H*, by acting on NPR3, a salicylic acid receptor (Ying et al., 2023). A recent study found that miR858b-MYB1L module plays a crucial role in Taxol biosynthesis by up-regulating the expression of *TBT* and *BAPT* genes in the endodermis (Yu et al., 2025a). Omics studies on miRNA-associated transcriptional and posttranscriptional regulation provide plenty of clues for enhancing Taxol biosynthesis (**Figure 3c**).

### 3.2 Genes involved in environmental stress responses

*Taxus* is a relict genus that dates back to the ancient Quaternary glacial period (Liu et al., 2023). The high economic value of Taxol has leaded to extensive illegal logging, pushing wild populations to the verge of extinction (Xiao-Lin and Xiao-Ri). Studying the genes related to environmental stress responses is a crucial strategy to improve the adaptability of *Taxus* populations.

UV radiation is a ubiquitous natural stressor that impacts the accumulation of active ingredients in *Taxus* plants (Zu et al., 2010). Transcriptomic analysis have shown that several genes involved in flavonoid biosynthesis, including *PAL1*, *CHS2*, *FLS1*, and *F3’5’H2*, are significantly up-regulated after UV-B exposure (Jiao et al., 2022). Combining RNA-seq with Internal Transcribed Spacer analysis highlighted the role of endophytic fungi in sex-specific responses to UV-B radiation, altering the expressions of various oxidation-reduction system-related genes, such as *APX2*, *GST7*, *NCED1*, *ZE1*, *CS1*, and *CM1* (Zhang et al., 2024). Transcriptomic data also revealed that multiple TF genes are induced by high light conditions, indicating their involvement in tolerance of *T. chinensis* to light or heat stresses (Li et al., 2022). Comparative proteomic analysis identified numerous proteins responsive to UV-A radiation, including forty-seven PSII proteins, six PSI proteins, one DXR, and one HDR (Zheng et al., 2016). Additionally, various omics studies have explored genes and proteins in *Taxus* that respond to environmental stresses like cadmium contamination, acid rain, nano-pollutants, intense light, high temperature, and cold stress (Hu et al., 2014; Meng et al., 2017; Majeed et al., 2020; Hu et al., 2022; Feng et al., 2023; Wang et al., 2025c). These omics findings offer numerous candidate genes and proteins that could be targeted to improve *Taxus* resistance to environmental challenges.

### 3.3 Genes involved in growth and development

To date, research on the growth and development of *Taxus* has primarily concentrated on aril color, seed germination, and the formation of adventitious roots. In three *T. mairei* specimens with three different color arils, metabolomic combined with transcriptomic analysis revealed significant differential expression of *HCT*, *LAR*, *ANS*, *NCED*, and *CCoAOMT* across the various aril colors (Yan et al., 2024). Integrating ATAC-seq and RNA-seq techniques revealed that the *C4H*, *CHS*, *F3H*, *DFR*, *PSY*, *PDS*, and *LUT1* genes are involved in the development of aril color in *T. mairei* (Yan et al., 2025). Multi-omics integration analysis identified many differentially expressed genes and differentially accumulated proteins during the seed germination process of *T. mairei* (Chen et al., 2023). Transcriptome sequencing showed that many hormone signaling-related genes undergo significant changes during adventitious root formation, which aids in enhancing the propagation and breeding of *Taxus* species (Ren et al., 2019). Additionally, combined transcriptome and metabolite analyses confirmed the involvement of hormones, amino acids, and secondary metabolites in rooting mediated by *A. rhizogene* (Wang et al., 2023).

## 4 Omics researches on biological compounds

### 4.1 High-throughput identification and analysis of taxanes

Taxanes, a group of natural compounds with nearly 600 known structures, are the primary bioactive components found in *Taxus* trees (Shi et al., 2025). With the advancement of detection technologies, dozens of new taxanes have been discovered, although their metabolic pathways remain unclear (Lange and Conner, 2021). Rapid and high-throughput analysis of these active ingredients, particularly the structurally diverse taxanes, has become a crucial part of *Taxus* research.

**Figure 4:**
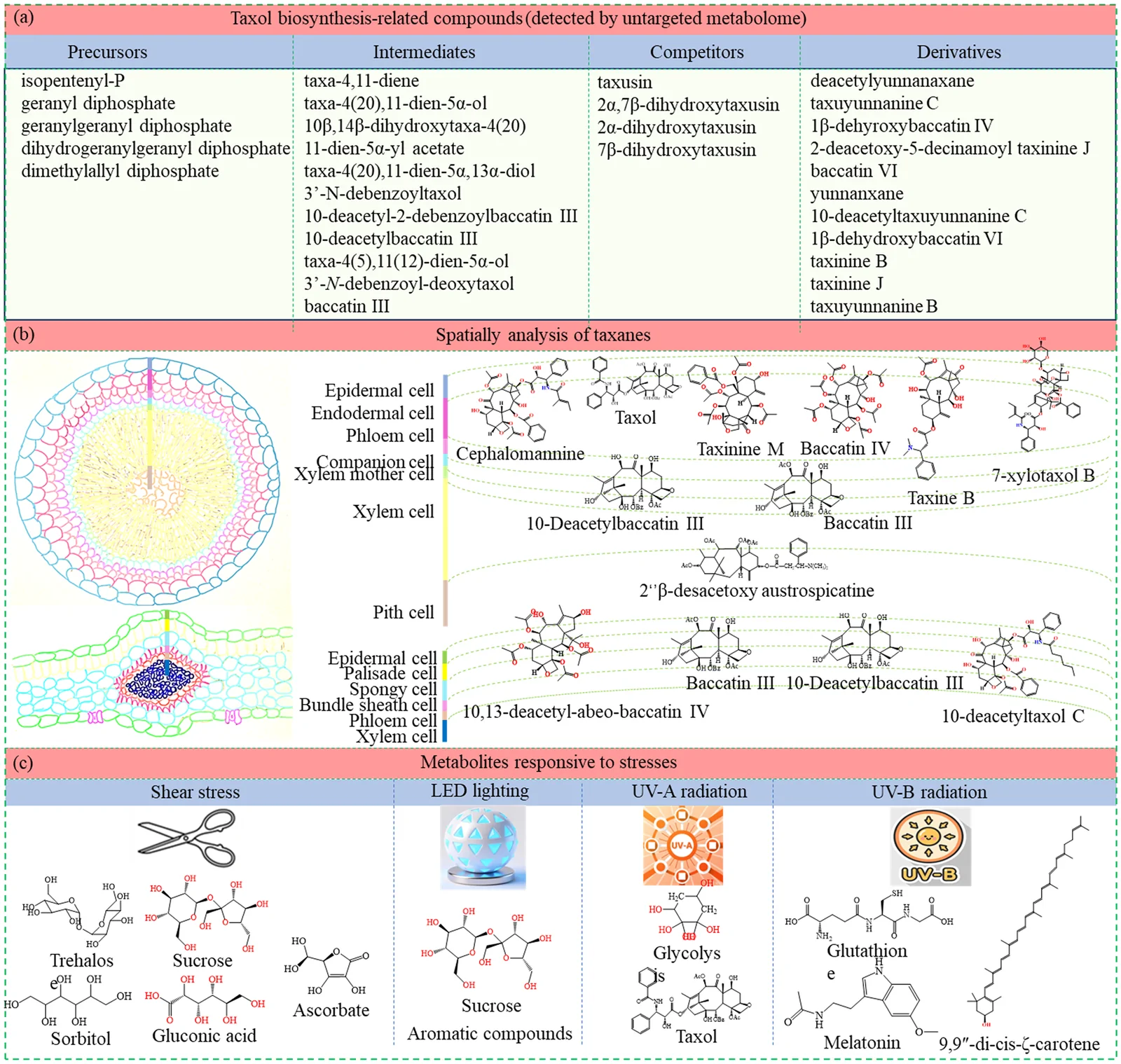
Omics researches on biological compounds of *Taxus*. **(a)** List of taxanes detectable by untargeted metabolomic analysis. **(b)** Spatially analysis of taxanes in the stems and leaves of *Taxus*. **(c)** Screening of metabolites responsive to stresses, including shear, LED lighting, UV-A radiation, and UV-B radiation.

Metabolomics was applied to *Taxus* before transcriptomics. In 2003, the first metabolomic analysis of *Taxus* was conducted using metabolites from *T. media* cell suspension cultures treated with MeJA (Ketchum et al., 2003). Because the compound database was incomplete, compound identification depended on existing taxoid standards. In that 2003 metabolomic study, 13 taxoids were identified, most of which were C-13 oxygenated taxoids (Ketchum et al., 2003). Since then, the metabolomics approach has been applied to reveal the metabolic profiling of various *Taxus* species, including *T. mairei*, *T. yunnanensis*, *T. chinensis*, *T. fuana*, *T. contorta*, *T. baccata*, and *T. cuspidata* (Tanaka et al., 2011; Yu et al., 2018; Zhou et al., 2019b; Majeed et al., 2020; Yu et al., 2021b; Perez-Matas et al., 2023a). Concerning the Taxol biosynthesis pathway, most compounds fall into three major categories: precursors, intermediates, and competitors (Croteau et al., 2006). By summarizing different metabolomic studies, the detectable compounds in each category were listed. Using conventional untargeted metabolomics, precursors, such as isopentenyl-P, geranyl diphosphate, geranylgeranyl diphosphate, dihydrogeranylgeranyl diphosphate, and dimethylallyl diphosphate, can be detected; intermediates, such as 3’-*N*-debenzoylTaxol, 10-deacetyl-2-debenzoylbaccatin III, 10-deacetylbaccatin III, 3’-*N*-debenzoyl-deoxyTaxol, and baccatin III can be detected; and competitors, taxusin and its hydroxylates can be detected (Yu et al., 2018; Zhou et al., 2019b). Besides, many Taxol derivatives, such as deacetylyunnanaxane, taxuyunnanine C, 1β-dehyroxybaccatin IV, 2-deacetoxy-5-decinamoyl taxinine J, baccatin VI, yunnanxane, 10-deacetyltaxuyunnanine C, 1β-dehydroxybaccatin VI, taxinine B, taxinine J, taxuyunnanine B, 2′β-deacetoxyaustrospicatine, 2-deacetoxytaxinine J, and 7, 2′-didesacetoxyaustospicatine, also can be detected using untargeted metabolomics (Tanaka et al., 2011; Song et al., 2014). Furthermore, an untargeted metabolomics study using HPLC-MS/MS based untargeted metabolomics identified eight main taxanes, including 10-deacetylTaxol, 9-dihydro-13-acetylbaccatin III, 10-deacetylbaccatin III, docetaxel, paclitaxel, cephalomannine, 10-deacetyl-7-xylosyl paclitaxel, and baccatin III (Bai and Li, 2024) (**Figure 4a**).

### 4.2 Spatial analysis of taxanes

Omics data regarding the spatial distribution of active compounds provide an indispensable guidance for sampling and assist in uncovering the functions of these components (Zou et al., 2025). The spatial distribution of active components within *Taxus* tissues has been a key area of research. For instance, the spatial distribution of taxanes in *T. media* has been documented (Soliman and Raizada, 2020; Yu et al., 2020). Recently, Mass Spectrometry Imaging (MSI), a powerful tool, has been developed to offer a label-free method for *in situ* localization of compound with high spatial resolution (Sumner et al., 2015). This advancement has made it possible to visually analyze taxanes in different *Taxus* tissues.

In 2017, Laser Desorption/Ionisation MS (LDI-MS) Imaging, combined with gold nanoparticle-enhanced target analysis, was employed to investigate *T. baccata*, visualizing that several taxanes, such as taxinine M, baccatin IV and taxine B, are predominantly localized in the cortex (Arendowski and Ruman, 2017). Subsequently, in 2019, an innovative matrix-enhanced *in situ* detection and imaging technique enabled the visualization of 111 low-molecular-weight compounds from germinating seeds of *T. mairei* (He et al., 2019). Although taxanes were not identified in this study, the method represents a significant advancement in compound visualization technology. More recently, in 2023, a novel matrix-assisted laser desorption/ionization mass spectrometry imaging (MALDI-MSI) approach was applied to map the distribution of multiple taxanes in the stems and leaves of *T. mairei*. Specifically, nine taxanes, including 10-deacetylpaclitaxel, 10-deacetylbaccatin III, baccatin III, Taxol, 2’-desacetoxy austrospicatine, 7-xyloTaxol B, 10, 13-deacetyl-abeo-baccatin IV, 10-deacetylTaxol C, and Taxol B, and four taxanes, including 10-deacetyl cephalomannine, 10-deacetylpaclitaxel, baccatin III, and 10-deacetyl baccatin III, were detected in the stem and leaf of *T. mairei*, respectively (Yu et al., 2023; Zhan et al., 2023a). Beyond taxanes, MALDI-MSI technology has also facilitated the visualization of a broad spectrum of other bioactive compounds, including flavonoids, steroids, coumarins, phenolics, terpenes, and glycosides (Zhan et al., 2024a, 2024b) (**Figure 4b**).

### 4.3 Identification of metabolites responsive to stress

Plants possess a remarkable capacity to synthesize a wide array of secondary metabolites, positioning them as exceptional natural chemists (Zhan et al., 2022). *Taxus* species are likely to employ distinctive chemical strategies to withstand environmental stressors. Metabolic profiling of *T. cuspidata* identified trehalose, sorbitol, ascorbate, sucrose, and gluconic acid as principal defense-related metabolites responsive to shear stress (Han and Yuan, 2009). Furthermore, 1H-NMR-based metabolomic analysis demonstrates that supplemental LED lighting significantly induced the accumulation of sucrose and aromatic compounds in *T. baccata* (Chiocchio et al., 2022). GC-MS-based metabolomic analysis revealed that UV-A radiation activates the stress defense responses in *Taxus* by upregulating pathways associated with photosynthesis, glycolysis, and secondary metabolism (Zheng et al., 2016). Concurrently, exposure to UV-B radiation enhanced the defense response by increasing levels of metabolites such as 9, 9″-di-*cis*-*ζ*-carotene, pre-squalene diphosphate, glutathione, and melatonin (Zhang et al., 2024). Additionally, a metabolomic study of *T. baccata* pollen grains identified 21 metabolites exhibiting differential accumulation under varying nitrogen and phosphorus deposition conditions (Pers-Kamczyc and Kamczyc, 2022). Future research integrating MSI techniques to spatially visualize metabolite dynamics in *Taxus* under environmental stress represents a promising avenue for advancing our understanding of their adaptive chemical responses (**Figure 4c**).

## 5 Technical progress in *Taxus* omic research

### 5.1 Increasing number of detectable genes and metabolites

Prior to the availability of genomes, transcriptomic studies primarily relied on *de novo* assembly methods to obtain reference sequences (Martin and Wang, 2011). The quantity of unigenes serves as a critical metric for assessing transcriptome quality and provides a foundation for subsequent comprehensive analyses of metabolic pathways. Early transcriptomic investigations conducted in 2011 utilized platforms such as Roche GS FLX Titanium and Illumina GA IIX, yielding 20, 557 and 36, 493 unigenes for *T. cuspidata* and *T. mairei*, respectively (Hao da et al., 2011; Wu et al., 2011). Subsequently, transcriptomes generated using the Illumina HiSeq™ 2000 platform reported increased unigene counts of 71, 479 and 88, 194 in *T. yunnanensis* and *T. media*, respectively (Yu et al., 2017). The expansion in unigene numbers facilitated the identification of candidate genes involved in the biosynthetic pathway of Taxol. Leveraging genomic data, draft genome assemblies for *T. mairei*, *T. wallichiana*, and *T. yunnanensis* revealed 42, 746, 44, 008, and 34, 931 protein-coding genes, respectively (Cheng et al., 2021; Song et al., 2021; Xiong et al., 2021). Since 2021, genome-based transcriptomic analyses have predominantly focused on profiling gene expression patterns rather than the discovery of novel functional genes.

The quantity of detectable metabolites plays a pivotal role in metabolomic analyses. Initial metabolomic studies were limited to identifying only a small number of compounds, encompassing merely a few taxanes. For instance, 65 intracellular metabolites were detected in *T. cuspidata* (2009), 10 compounds in *T. mairei* (2011), and 12 metabolites in *T. chinensis* (2014) (Han and Yuan, 2009; Tanaka et al., 2011; Song et al., 2014). However, technological advancements have significantly expanded the scope of detectable metabolites, with recent untargeted metabolomic approaches enabling the detection of thousands of compounds (Yu et al., 2018; Zhou et al., 2019b). Currently, untargeted metabolomics facilitates the comprehensive identification and quantitative analysis of a wide array of compounds, including taxanes.

### 5.2 Visualization of omics data on *Taxus*

#### 5.2.1 Visualized gene expression pattern

Various visual omics methodologies, including single-cell transcriptomics, spatial transcriptomics, and laser capture microdissection (LCM)-based proteomics have been employed to investigate secondary metabolism in medicinal plants (Liu et al., 2024; Wang et al., 2024b; Wu et al., 2025; Zhan et al., 2026). While several bulk RNA sequencing studies have revealed gene expression patterns in *Taxus* under diverse conditions, profiling gene expression at the level of individual *Taxus* cell offers more direct insights into regulatory relationships, as opposed to inferred interactions. Utilizing tissue-specific marker genes, a single-cell atlas of *Taxus* was constructed, revealing spatial expression patterns within leaf and stem tissues (Yu et al., 2023; Zhan et al., 2023a). Visual analyses demonstrated that the majority of genes associated with Taxol biosynthesis were predominantly expressed in the epidermal, endodermal, and xylem tissues of the stem, as well as in the mesophyll cells of the leaf (Yu et al., 2023; Zhan et al., 2023a). Additional analyses indicated that genes involved in phenolic acid and flavonoid biosynthesis exhibited significant expression in leaf epidermal cells (Zhan et al., 2024a). The regulation of the biosynthesis of secondary metabolites in medicinal plants is orchestrated by a complex TF-target network (Perez-Matas et al., 2023b). Previous identification of *Taxus* TFs relied on bulk transcriptomic data, which may have introduced false positives. In contrast, visualization-based analyses have introduced a novel criterion for TF screening, emphasizing the concordance of spatiotemporal expression patterns between TFs and their target genes, thereby substantially enhancing the precision of predicting transcriptional regulatory modules.

#### 5.2.2 Visualized compound accumulation pattern

The visualization of chemical compounds has garnered considerable attention within the research community. Histochemical studies have been applied to determine the characteristics of the formation and localization of phenolic compounds with high biological activity in various organs of plants of the genus *Taxus* (Zaytseva et al., 2026). The spatial distribution patterns of various taxanes and terpene alkaloids were visualized in the stem of *T. baccata* through MS imaging enhanced by gold nanoparticles (Arendowski and Ruman, 2017). Similarly, Cryo-TOF-SIMS/SEM combined with microscopic imaging techniques visualize the distribution patterns of taxanes in the stems of *T. cuspidata* (Gong et al., 2024). Furthermore, MALDI-MSI analysis has been utilized to map the distribution of taxoids across the stems and leaves of *T. mairei* (Yu et al., 2023; Zhan et al., 2023a, 2024). In addition, the spatial dynamics of lipids during seed germination in *T. mairei* were visualized using MALDI-MSI technology (Chen et al., 2023). This emerging MS-based imaging approach enables both qualitative and quantitative investigations of plants by providing detailed spatial molecular distributions. However, MALDI-MSI analyses often suffer from high mass errors attributable to calibration biases or limited mass spectrometer resolution. Therefore, compound identification should be corroborated through complementary fragmentation data, such as MS/MS or nuclear magnetic resonance (NMR) spectroscopy.

### 5.3 Integration analysis of multiple omics

In woody plants, conducting mutant screening and genetic transformation experiments presents significant challenges (Ketchum et al., 2007; Perez-Matas et al., 2023b). Consequently, the application of multiple omics approaches, such as transcriptomics, proteomics, and metabolomics, have emerged as a valuable strategy for investigating the biological complexities of *Taxus* species. The integration of these diverse omics datasets facilitates a more comprehensive understanding of underlying biological mechanisms (Pan et al., 2025).

**Figure 5:**
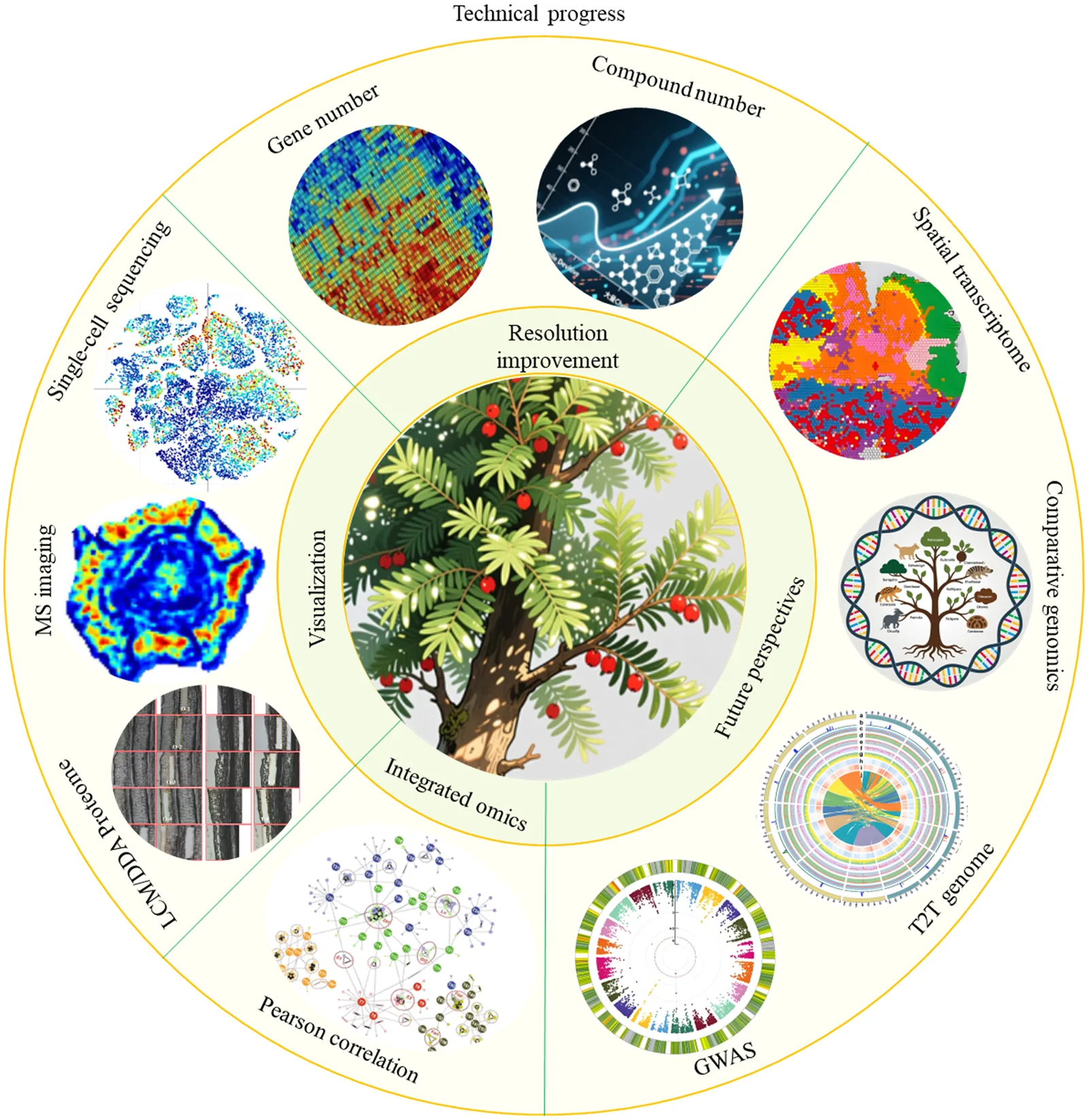
Technical progress in *Taxus* omic research.

The integration transcriptomic and metabolomic data can facilitates a comprehensive examination of the substrate-enzyme-product modules at each step of the Taxol biosynthesis pathway (Yu et al., 2021b, 2022). Such integrative analyses enable researchers to determine the rate-limiting steps within Taxol biosynthesis and to screen rate limiting enzymes (Zhang et al., 2023; Jiang et al., 2025). Additionally, integrated transcriptome and metabolome analyses have been instrumental in elucidating various biological phenomena, such as interspecies variation, sexual dimorphism, environmental stress resistance, and pigment formation in arils (Yu et al., 2022; Yan et al., 2024; Wang et al., 2025c). Recently, Shen and colleagues employed integrated transcriptomic and metabolomic approaches to uncover potential mechanisms underlying the sex-specific responses to ultraviolet UV-B radiation and fungal infection, and age-related responses of twigs to fungal elicitor infection (Zhang et al., 2024; Wang et al., 2025a; Zhan et al., 2025). Based on the differentially expressed genes and differentially accumulated metabolites, Pearson correlation analysis predicted several putative transcriptional regulators implicated in the modulation of secondary metabolism. Notably, MYC2 was recognized as a key factor responsive to fungal elicitor infection; additionally, four TFs (GATA, bHLH, MYB, and NAC) were associated with responses to nano-pollutants, and a MYB gene (KI387_007149) exhibited strong expression in yellow arils, all identified via Pearson correlation analysis (Yan et al., 2024; Wang et al., 2025a, 2025). Omics correlation analyses further elucidate complex internal regulatory networks governing Taxol and its precursor biosynthesis (Song et al., 2014). Integrated proteomic and metabolomic analyses, investigated the defense responses of *T. chinensis* to a short-term UV-A radiation (Zheng et al., 2016). In cultivated *T. media*, the chemical composition and protein accumulation of various major stem cell types was analyzed by an integrated metabolomic and proteomic method (Yu et al., 2020). Moreover, single-cell ATAC-seq combined with transcriptomic data revealed a strong correlation between chromatin accessibility peaks and the expression of Taxol biosynthesis-related genes (Zhan et al., 2023b) (**Figure 5**).

## 6 Future perspectives

The genus *Taxus* represents a distinctive group of gymnosperms renowned for their remarkable botanical longevity, cultural importance, and notable pharmacological potential (Jîjie et al., 2025; Shi et al., 2025). The review explores the utilization of omics approaches in *Taxus* research, encompassing investigations into genetic diversity, gene function, and metabolic engineering.

With ongoing technological advancements, the application of diverse omics methodologies to the study of *Taxus* species is expected to expand. Firstly, with the emergence of DNA sequencing technologies, the era of telomere-to-telomere (T2T) genome assemblies is approaching (Huang et al., 2025). Although several *Taxus* genomes have been published, most of them are assembled at the chromosome level rather than at the T2T level (Cheng et al., 2021; Song et al., 2021; Xiong et al., 2021). Consequently, the identification of certain key enzymes involved in the Taxol biosynthesis pathway remains incomplete (Wang et al., 2025b). Recently, the first T2T genome of the *Taxus* genus (*T. wallichiana*) was published, identifying a batch of 2-oxog lutarate/Fe(II)-dependent dioxygenases, suggesting that generating of T2T genome sequences may provide critical insights to address these gaps. Secondly, spatial transcriptome sequencing has been applied in crop species, but it has only recently been used in the study of woody plants (Liu et al., 2022; Xia et al., 2022; Li et al., 2023). Due to technical challenges such as tissue slice detachment, spatial transcriptomics in woody species is still in its infancy; to date, only the spatial transcriptome of poplar young stems has been reported (Du et al., 2023). Successful application of spatial transcriptomics to *Taxus* could yield significant breakthroughs in elucidating the Taxol biosynthesis pathway. Lastly, comparative genomics analyses are warranted (Wang et al., 2025b). Comparative studies between *Taxus* and closely related taxa, including *Torreya grandis* and *Pseudotaxus chienii*, have the potential to illuminate the origin and evolutionary of the taxane biosynthesis pathway (Lou et al., 2023). Collectively, these developments mark the inception of a new era in *Taxus* research, driven by the integration of advanced omics technologies.
